# Long-Lasting Insecticide-Treated Nets: Assessment of the Awareness and Utilization of Them among Antenatal Clinic Attendees in Abakaliki, Southeast Nigeria

**DOI:** 10.1155/2020/2984867

**Published:** 2020-04-30

**Authors:** Chidebe Christian Anikwe, John Chiadikobi Irechukwu, Bartholomew Chukwunonye Okorochukwu, Cyril Chijioke Ikeoha, Johnson Akuma Obuna, Brown Nnamdi Ejikeme, Ifeyinwa Helen Anikwe

**Affiliations:** ^1^Department of Obstetrics and Gynaecology, Federal Teaching Hospital Abakaliki, PMB 102, Abakaliki, Ebonyi State, Nigeria; ^2^Department of Obstetrics and Gynaecology, Federal Medical Centre, Owerri, P.O. Box 1010, Owerri, Imo State, Nigeria; ^3^Department of Administration, Federal Teaching Hospital Abakaliki, PMB 102, Abakaliki, Ebonyi State, Nigeria

## Abstract

**Background:**

The use of long-lasting insecticide-treated nets (LLITNs) is one of the effective strategies for the prevention of malaria, especially among pregnant women.

**Aim:**

This study is aimed at assessing the awareness and utilization of LLITNs during pregnancy among antenatal clinic attendees at the Alex Ekwueme Federal University Teaching Hospital Abakaliki.

**Materials and Methods:**

This was a cross-sectional study among antenatal attendees at the Alex Ekwueme Federal University Teaching Hospital Abakaliki, Ebonyi State. A semistructured questionnaire was used to obtain relevant information from the participants. Data analysis was done using SPSS version 20.

**Results:**

The mean age of the women was 26.05 ± 5.76 years. About one-third (30%) of the respondents were nulliparous. Most of the respondents had at least a secondary education. More than ninety percent of the respondents had a good knowledge of malaria with 95.8% being aware of LLITNs. The main source of information was from hospitals (54.5%). The rate of utilization of LLITNs was 37.5%; however, consistent use was only reported by about a third of this proportion. The major reasons for not utilizing the nets include discomfort/heat and fear of the chemical content. Women with tertiary education were more likely to utilize mosquito nets during pregnancy compared with women with secondary or primary education. Women who live in rural areas (OR = 0.393 95% CI 0.602–0.073) were less likely to use LLITNs during pregnancy, while those who are aware of the aetiology of malaria (OR = 4.38 95% CI 0.983–19.591) were more likely to utilize LLITNs in pregnancy.

**Conclusion:**

The level of awareness of LLITNs is high; however, its utilization was discouragingly low. Rural dwellers and those without appropriate knowledge of the aetiology of malaria were less likely to use LLITNs in pregnancy.

## 1. Introduction

Malaria is a major public health problem in tropical and subtropical regions of the world, especially among pregnant women because of the associated maternal and perinatal morbidity and mortality [1]. Most of the cases in 2016 were in the World Health Organization (WHO) African Region (90%), followed by the WHO South-East Asia Region [2]. The WHO World Malaria Report of 2017 has demonstrated the enormity of the burden of malaria with an estimated 219 million cases of malaria occurring worldwide with an estimated 435,000 deaths from malaria globally, compared with 451,000 estimated deaths in 2016 and 607,000 in 2010 [3]. The burden of malaria is largely borne by Africa; Nigeria accounted for the highest proportion of cases globally (27%), followed by the Democratic Republic of the Congo (10%), India (6%), and Mozambique (4%) [2]. Each year, there are an estimated 25 million pregnancies in sub-Saharan Africa [3] and an estimated 8.7 million pregnancies in Nigeria which are at risk of malaria with malaria-related complications contributing to about 11% of the maternal mortality in Nigeria [1, 4]. Malaria in pregnancy is a formidable misery in sub-Saharan Africa. It has been estimated that the prevalence of malaria attacks during pregnancy is significantly lower in high-intensity transmission areas than it is in areas of medium or low intensity [5]. Pregnant women are a special group at high risk of malaria infection and the prevention of malarial attacks in this group is important. This will help in reducing some of the complications of malaria in pregnancy such as early pregnancy loss, anaemia in pregnancy, preterm births, intrauterine growth restriction, stillbirths, and perinatal mortality [6, 7]. Apart from these, its prevention will help in reducing its significant contribution to human and economic wastage [8, 9].

World Health Organization Initiative in malaria prevention and control such as the Roll Back Malaria stipulated the use of long-lasting insecticide-treated nets (LLITNs) as one of the key strategies for malaria prevention and control in sub-Saharan Africa [6, 10]. The evidence for the efficacy of LLITNs in preventing malaria in pregnancy has been well documented, and a strong correlation between the use of ITNs and the improvement of fetal-maternal outcomes has been established [11–14]. This fetomaternal outcome variable includes reduction of perinatal rate, intrauterine growth reduction, and reduction in the burden of *Plasmodium* parasitemia and the number of pregnancies complicated by anaemia. A long-lasting insecticide-treated net (LLTIN) is a factory-treated mosquito net made with a netting material that has insecticide incorporated within or bound around the fibers [15] which is expected to retain its effective biological activity without retreatment for at least twenty WHO standard washes and three years of recommended use under field conditions [16]. Newer LLITNs made from polyester nets and propylene nets can last 2-3 years and 5–7 years, respectively [10, 17]. The Roll Back Malaria (RBM) program recommended 60% utilization of LLITNs towards the attainment of the Millennium Development Goals (MDGs) 4, 5, and 6 [7] (now Sustainable Development Goal 3) [18]. The use of LLITNs is very effective in the prevention and control of malaria. When properly used, LLITNs can reduce malaria transmission by at least 60% [8]. Therefore, malaria control with the use of LLITNs has been recommended at all levels as an important component of antenatal care and WHO has been at the forefront of sponsoring the distribution of LLITNs in malaria-endemic areas [8]. It is worrisome that despite the concerted efforts made in the provision of LLITNs, distribution and use of these nets remains a challenge among pregnant women. A qualitative systematic review of the uptake of malaria prevention interventions in pregnancy in Africa found out that cost, ignorance, distance to health facilities, and other factors influence women's uptake of these interventions [8]. Other reasons for nonuse include discomfort, use of other preventive measures, and perceived low mosquito density [9].

**Figure 1:**
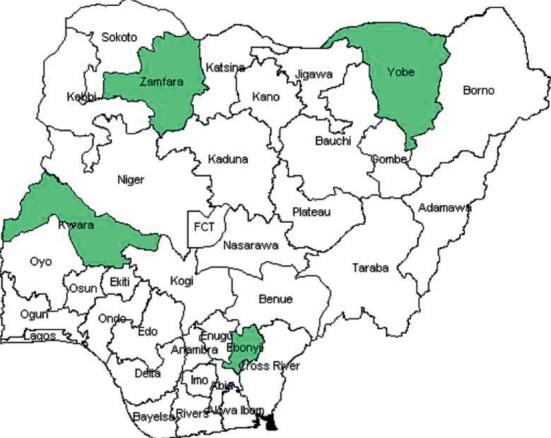
ITN coverage in Nigeria.

The use of LLITNs is one of the strategies of RBM initiative adopted in the Abuja Declaration of African Summit in April 2000 in which African Regional leaders expressed commitment to ensuring that 60% of pregnant women in malaria-endemic areas have access to effective malaria preventive services by the year 2005 [5, 7, 9]. This form of vector control measure is very effective in the control of malaria in pregnancy. Different studies in sub-Saharan Africa have found increased awareness of the importance of LLITNs as one of the malaria preventive measures. This is true as shown by the study of Njoroge et al. in Kenya where 86% of respondents had adequate knowledge of malaria and LLITNs [19], which is similar to a study in Uganda that gave a figure of 77% [20]. In Nigeria, various studies have identified increased awareness of LLITNs by pregnant women. The level of awareness ranges from 74% in northwestern Nigeria to 92% in the southeastern part of the country [1, 4, 21, 22]. This increased awareness suggests that a lot of health education has taken place in sensitizing pregnant women to the importance of LLITNs as it is one of the key interventions in the prevention and control of malaria. However, despite increased awareness amongst pregnant women, the utilization of LLITNs in sub-Saharan Africa is still low [20]. Similar low results have been reported in most studies in Nigeria [1, 9, 22, 23] which falls short of the global body target of 80% utilization of LLITNs among the population at risk. In the study by Ankomah et al. involving 2348 pregnant women in Nigeria, less than one-third of the women-owned insecticide-treated nets (ITNs) with only 25.7% of all pregnant women who owned ITNs is sleeping under a net [24]. Some of the reasons that could explain the low use of ITNs include discomfort, fear of suffocation, low awareness of need, misuse (hung over windows, used as a blanket), lack of awareness of its importance and its effectiveness, believe in seasonal nature of mosquito activity, its availability, and misconception [4, 25–27]. Other factors include women's parity, educational level, family size, marital status, social class, and place of residence [4, 24, 25, 27, 28]. Okonta [23] noted that women carrying their first pregnancy are significantly associated with the increased use of LLITNs which might be attributable to nulliparous women being more inclined to heed instructions given during the antenatal visits which can be attributed to the effect of the first experience. This finding fails to agree with the work of Akaba et al. [27], where usage of LLITNs is statistically related to increasing parity. This finding could be attributed to the increased knowledge and awareness garnered by these women via antenatal classes and immunization programs as a result of repeated exposure to information concerning malaria [20]. It has also been observed that the mother's level of education, educational class of the woman, and husband's educational level were significantly associated with the use of LLITNs [7, 22, 23, 27]. Generally, education comes with increased awareness of several health issues and also a better motivation to practice a healthy lifestyle. Akaba et al. [27] found out that discomfort, especially due to heat and reduced ventilation, size not fitting to bed, and nonacceptability by the husbands were the main reasons for low use among their study population. This was similar to the study by Isah and Nwobodo [22] but different from the study of Obol et al. [20] in Uganda where the far distance of the health centre and being single, widowed, or divorced hindered its utilization. The Roll Back Malaria initiative recommended that by 2010 at least 80% of people at risk of malaria (including pregnant women) use insecticide-treated bed nets (ITNs) in areas with the stable transmission. It is unfortunate that out of 5.8 million pregnancies estimated to have occurred in Nigeria in 2012, only 60.9% of these women were protected by ITNs and only 39.7% received intermittent preventive treatment for malaria [29]. With the Abuja target of 2005 having elapsed and attainment of the 2010 time set by the World Health Assembly in 2005 for the 80% realization of coverage targets also not reached in most states in Nigeria–(Figure 1) [29], a closer assessment of the factors that are pivotal to the realization of the target of RBM is extremely important. This study is, therefore, aimed at determining the awareness and utilization of this evidence-based malaria preventive strategy among antenatal clinic attendees at the ALEX EKWUEME Federal Teaching Hospital, Abakaliki.

## 2. Materials and Methods

### 2.1. Study Area

The study was carried out at the Alex Ekwueme Federal University Teaching Hospital Abakaliki (AE-FUTHA), Ebonyi State, between the 1st March and the 30th of June 2018. The hospital was established in December 2011 after a successful merger and acquisition of Ebonyi State University Teaching Hospital and the Federal Medical Centre, Abakaliki by Federal Government. It is a referral centre and the only teaching hospital in the state. It has 11 clinical departments of which Obstetrics and Gynaecology is one of them. The other clinical departments include Paediatrics, Internal Medicine, Surgery, Psychiatry, Community Health, Family Medicine, Radiology, Ophthalmology, ENT, and Anaesthesiology. The Obstetrics and Gynaecology department has ten teams, each comprising consultants, senior registrars, registrars, senior house officers, and house officers. The department runs Gynaecology, Antenatal, Family Planning, and Postnatal clinics daily. Nurses, pharmacists, and laboratory scientists also carry out stipulated roles in every clinic. The government of Ebonyi State through the Roll Back Malaria (RBM) initiatives distributes LLITNs to each household and each antenatal attendee in the hospital is provided with LLITNs and counseled to sleep regularly under it to help reduce the burden of malaria.

### 2.2. Study Design

This was a descriptive cross-sectional study among consenting pregnant women attending antenatal clinics using semistructured self-administered questionnaires. The questionnaires were pretested for clarity, assessment of the length of time for administration, comprehension, and other attributes. The questionnaire assessed the sociodemographic characteristics of the respondents, knowledge about malaria, and its preventive methods. Determinants of the utilization of long-lasting insecticide-treated nets were also assessed. Pregnant women included in the study were women that had access to LLITNs in their household and therefore were likely to use them. By household access, we mean proportion of households with one LLITN for every two people and the proportion of women with access to LLITNs within the household with the assumption that LLITNs protect on average two people [30]. The filled-out questionnaires were validated on-site for completeness.

### 2.3. Sample Size (*N*)

The minimum sample size (*N*) was calculated using the formula

(1) $$\begin{array}{c} N=\frac{Z^{2}pq}{e^{2}}, \end{array}$$

where *Z* is standard deviation at 1.96 (which corresponds to 95% confidence interval). *p* = 0.5 is the probability of the event occurring. *q* = 1−*p* = 0.5 is the probability of the event not occurring. *e* is the desired level of precision, also known as sampling error: 5% = 0.05. *N* = (1.96)^2^ × 0.5 × 0.5/(0.05)^2^ = 384.16.

Attrition rate of 10% was added to the sample size; hence, the final sample size was 384.16 + 38.41 = 422.6 approximately 423.

A total of 423 questionnaires were distributed among the antenatal attendees, but 400 were retrieved. Informed consent was obtained from all respondents before being interviewed. Respondents were selected from consecutive consenting pregnant women that presented for booking at the Antenatal Clinic each day. Women on return visits and those in need of urgent medical attention or already in labour were all excluded.

### 2.4. Data Analysis

Data collected was analyzed using a predesigned questionnaire. Descriptive analyses of the variables were done using Epi Info software version 7. The results were presented in frequency tables, charts, and contingency tables. Categorical data were presented as frequency and percentages, while continuous variables were presented as mean ± SD. Chi-square was used to determine the association of some sociodemographic characteristics and utilization of LLITNs. A *p*-value of <0.05 was considered statistically significant.

### 2.5. Ethical Consideration

Permission to carry out this research was sought and obtained from the Research and Ethics Committee of the Alex Ekwueme Federal University Teaching Hospital Abakaliki. The ethical approval reference number is FETHA/REC VOL *l*/2017/562. Informed written consent was obtained from the study population before the administration of the questionnaire.

## 3. Results

Four hundred and twenty-three questionnaires were distributed, but 400 were properly filled giving a response rate of 94.6%. As shown in Table 1, the mean age of the respondents was 26.05 ± 0.076. Most of the clients were aged between 20 and 34 years 329 (82.2%), multiparous 185 (46.3%), and married 369 (92.3%) and had at least secondary education as their highest educational qualification. Two hundred and forty (60%) lived in the urban area of the state. The majority of the study population were Igbos and Christianity was the predominant religion. Most of the women were civil servants.

Table 2 shows the knowledge of malaria and its adverse effects on pregnancy. Three hundred and eighty-four clients (96%) knew that malaria can be transmitted by mosquito bites, while others were not aware of the aetiology of malaria. Three hundred and fifty-two (88%) were aware that malaria can be harmful to the developing fetus. Two hundred and twenty-three (55.8%) knew that mosquito net can be used to prevent contact with mosquitoes, thus preventing malaria, and others pointed out other methods of prevention.

Three hundred and eighty-three respondents (95.8%) were aware of the LLITNs, while only 17 (4.2%) were not aware. More than half of the clients got information about LLITNs from health facilities. Interestingly, more than seventy percent of the respondents were not aware that the net could be retreated. Among the study participants, 150 (37.5%) sleep under LLITNs in the index pregnancy, while the remaining do not. Among those who sleep under the net, 49 (32.7%) do so regularly, while 101 (67.3%) used it occasionally. Factors affecting their utilization among our respondents include discomfort/heat 160 (40%), fear of the chemical 7 (1.8%), using other measures 36 (9%), and not acceptable to husbands 8 (2%) as shown in Table 3.

**Figure 2:**
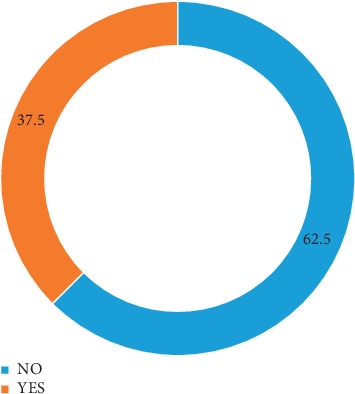
Pie charts showing the proportion of respondents who use LLITNs in the index pregnancy.

The pie chart shows the proportion of respondents that utilize LLITNs in the index pregnancy. From the chart, 37.5% of the respondents use LLITNs in the index pregnancy, while 62.5% did not use them for one reason or another (Figure 2).

States shown in green (Figure 1) met the RBM target of 80% coverage of ITN [29].

Table 4 shows a cross-tabulation of some sociodemographic characteristics and knowledge of the aetiology of malaria and utilization of long-lasting insecticide-treated nets. The table showed that the age and the parity of the respondents were not a significant determinant of LLITNs utilization. However, women with tertiary education were more likely to utilize mosquito nets during pregnancy compared with women with secondary or primary education. Women who live in rural areas were less likely to utilize LLITNs in pregnancy relative to urban dwellers (OR = 0.393 95% CI 0.602–0.073). Also, those who were aware that mosquitoes cause malaria were more likely to utilize LLITNs in pregnancy compared with those who are aware of the aetiology of malaria (OR = 4.38 95% CI 0.983–19.591).

## 4. Discussion

The proportion of antenatal clinic attendees that were aware of long-lasting insecticide-treated nets in this study was 95.8% which is in keeping with high awareness level that was reported in various studies in Nigeria [1, 30–32]. This finding from this study and other studies in Nigeria suggests that a lot of health education has taken place on the importance of LLITNs in reducing the contribution of malaria to maternal and under-five mortality in Nigeria. However, lower levels of awareness have been reported in other parts of Nigeria and outside Nigeria with various studies quoting a range of 64–77% [19, 23]. This difference in the level of awareness may not be unrelated to the characteristics of the study participants as the majority of the women in this study had tertiary education as their highest level of education which has earlier been documented by Omaka-Omari and Nwimo [33] as a significant contributor to pregnant women having good knowledge of malaria in the study area. Credence to the influence of maternal level of education on improving knowledge and acceptance of malarial interventions programs has also been reported by Singh et al. [6] and Hill et al. [25]. Omaka-Omari and Nwimo [33] on their study which assessed the pregnant women's malaria knowledge in Ebonyi State reported that urban dwellers have a better knowledge of malaria which is also evident in our study. Rural dwellers in our study have a highly significant increased odds of not using LLITNs, despite having access to it, when compared to the urban pregnant women (OR = 0.60 95% CI 0.39–0.92) despite probably being at higher risk of malarial attack. This is a worrisome finding in the study and calls for a massive education of rural dwellers on the importance of using LLITN during pregnancy and afterward. This education is important in reducing the observed misconception of malaria among pregnant women in the state [33]. Improvement of knowledge among the women is generally likely to increase the use of ITNs in their household, thereby reducing the attacks of malaria especially among the under-five.

Insecticide-treated net is one of the evidence-based approaches in reducing the burden of malaria among a population at risk [7], but its usage in sub-Saharan Africa of 54% by people at risk is far from the global goal of universal access [2]. In a review on the ownership and use of insecticide-treated nets during pregnancy in sub-Saharan Africa, Singh et al. reported that the rate of ownership varies from 3% to more than 80% across the region with a great discrepancy between ownership and usage [6]. Singh et al. reported low usage and the top reasons affecting use include women's expression of discomfort following sleeping under ITNs, poor understanding of their importance and proper usage [6]. However, it has been noted that the use of household ownership of ITNs to access usage might not be idle because of insufficient intrahousehold net saturation factor [34]. Koenker and Kilian [34] reported that population access to ITNs is accompanied by increased use, thus de-emphasizing household ownership in determining ITNs use. Despite the impressive good knowledge of long-lasting insecticide-treated nets among our respondents, their utilization was abysmally low which is in tandem with earlier reports [6, 35]. In the index study, only 37.5% of the respondents were using ITN with only 32.7% consistently using it. This figure is rather too low considering that this primary preventive method is very effective in preventing malaria in pregnancy, and this results in showing that owning LLITN does not always translate to its utilization [4, 6]. This low level of utilization had been reported in other similar studies; Isah et al. [22] reported utilization level of 13 %, while Ugwu and coworkers reported 39.1% [7]. Ozims et al. [1] reported 52% utilization among their study respondents [1]. This trend had also been observed in similar studies outside Nigeria in which 20.3–35% utilization rates had been reported [19, 20]. Despite the frantic effort made by the Nigerian government and nongovernmental organizations to provide information about the nets and provision of the nets at no cost, the level of impact still leaves room for much work, especially with regard to myths and misconception regarding the use of LLITNs [3, 6, 18, 36].

Among those who do not use the ITNs in our study, the commonest reason adduced was “discomfort in sleeping under the net”; this was also the commonest reason noted for nonutilization of the net in similar studies [1, 7, 22]. In poorly aerated rooms, the ambient room temperature may be elevated and this may cause discomfort to the pregnant women. This may be a particularly important concern in Nigeria where there is an epileptic power supply, leading to poor aeration of the rooms. The two other common reasons given by our respondents are that they are using other measures to prevent contact with mosquitoes and that hanging of mosquito net makes the room untidy; this trend had been observed by other researchers [36]. This finding calls for more enlightenment programs to be initiated in the study area to help correct these findings. The age and the parity of the respondents were not a significant determinant of LLITNs utilization. However, women with tertiary education were more likely to utilize mosquito nets during pregnancy compared with women with secondary or primary education. They are more likely to get the proper information about the causes of malaria in pregnancy and its prevention. Other studies had demonstrated this relationship [7, 22]. The place of the resident was also an important determinant of the use of insecticide-treated nets in this study. Women who live in rural areas were less likely to utilize LLITNs in pregnancy relative to urban dwellers. This might be attributed to the influence of education and information received by urban dwellers on positively directing their attitude and practice towards strategies aimed at the prevention of malaria [6]. Correct knowledge is an important driver for change [36]. Respondents who were not aware that mosquitoes cause malaria were less likely to utilize LLITNs in pregnancy compared with those who are aware of the aetiology of malaria. Knowledge of the adverse outcomes of malaria in pregnancy may be another motivation to the consistent use of LLITNs in pregnancy. Hence, one of the strategies needed to increase utilization of LLITN in pregnancy is to give appropriate information to the aetiology and adverse pregnancy outcomes associated with malaria in pregnancy.

### 4.1. Limitation of the Study

One of the limitations of this study is that it is not possible to determine causal relationships but only to test for associations because of the cross-sectional nature of the study. There may have been recalling bias and some respondents may have been reluctant to disclose an experience of LLITN use or might have exaggerated experience because of social desirability bias. An effort was, however, made to reduce these errors by encouraging the respondent to recall LLITNs use in the last one year. The respondent was educated on the study instrument and the study instrument was pretested among 30 pregnant women checking understanding and time needed for the proper answering of the questionnaire. Our study, however, provides useful information that could guide obstetricians, hospital managers, and policymakers on how to increase utilization of LLITNs among the obstetric population.

In conclusion, this study has shown the high level of awareness of LLITN among the study population which is not commiserated with the level of use of the net. The significant determinant of the use of ITNs in our study is the maternal level of education, place of residence, and appropriate knowledge of the role of the mosquito in the development of malaria. The reasons given for not sleeping under LLITNs are not acceptable. To help improve uptake and use of ITNs, pregnant women especially those in the hinterland should be educated via antenatal classes about malaria, its pathogenesis and vector control necessary in reducing the attack of malaria. Government of Ebonyi State should also assist by the provision of ITNs free or at a subsidized rate to encourage ownership, and it is also paramount that the Community Health Extension Workers (CHEW) be sent to the villages to help educate the general public, especially women, on the importance of owning and using ITNs in their household.

## Figures and Tables

**Table 1:** Sociodemographic status of respondents.

Parameter	Frequency	Percentage
Age		
<20	35	8.8
20–34	324	82.2
≥35	36	9.0

Parity		
0	120	30.0
1–4	185	46.3
≥5	95	23.7

Marital status		
Single	14	3.5
Married	369	92.3
Divorced/separated	9	2.2

Educational qualification		
Primary	61	15.3
Secondary	243	60.7
Tertiary	96	24.0

Residence		
Rural	160	40.0
Urban	240	60.0

Ethnicity		
Igbo	180	95.0
Hausa	5	1.3
Yoruba	10	2.5
Others	5	1.3

Religion		
Christianity	379	94.8
Islam	21	5.2

Occupation		
Farming	24	6.0
House wife	95	23.8
Trading	114	28.5
Civil servant	167	41.8

Husband occupation		
Nonskilled	140	35.0
Semiskilled	151	37.8
Skilled	109	27.2

**Table 2:** Knowledge about malaria.

	Frequency	Percentage
Is malaria caused by mosquitoes?		
Yes	384	96.0
No	12	3.0
I do not know	4	1.0

Is malaria harmful to the fetus?		
Yes	352	88.0
No	8	8.0
I do not know	40	40.0

Malaria can be prevented by		
Window and door nets	100	25.0
An indoor spray of insecticides	65	16.3
Mosquito nets	223	55.8
Antimalarial drugs	12	3.0

**Table 3:** Level of utilization of long-lasting insecticide-treated nets.

	Frequency	Percentage
Are you aware of long-lasting insecticide-treated nets?		
Yes	383	95.8
No	17	4.2

What is the source of information?		
Radio	61	15.3
Television	82	20.5
Church	39	9.8
Hospital	218	54.5

Can the net be retreated?		
Yes	100	25.0
No	29	7.3
I do not know	271	67.8

How often do you sleep under the LLITN? (150)		
Every day	49	32.7
Occasionally	101	67.3

Why do not you sleep under LLITN? (250)		
Discomfort/heat	160	64.0
Fear of the chemical	10	4.0
I use other measures	36	14.4
Not acceptable to husband	8	3.2
Makes the room untidy	25	10.0
No mosquitoes in the room	7	2.8
Does not prevent mosquito bites	4	1.6

**Table 4:** Factors associated with the increased use of LLITNs by mothers.

Factors	Used LLITNs	Did not use LLITNs	Total	Chi-square (X^2^)	*p* value
Age				0.833	0.2614
<20	16	19	35		
20–34	120	209	329		
≥35	14	22	36		

Parity				3.228	0.072
Nullipara	48	72	120		
Multipara	61	124	185		
Grand multipara	41	54	95		

Educational				9.926	0.002^*∗*^
Not formal/primary	24	37	61		
Secondary	75	168	243		
Tertiary	51	45	96		

Residence				5.358	0.020^*∗*^
Rural	49	111	160		
Urban	101	139	240		

Does mosquitoes' bite lead to malaria?				4.430	0.035^*∗*^
Yes	148	236	384		
No	2	14	16		

^*∗*^ *p* < 0.05 = statistically significant.

## Data Availability

The data used to support the findings of this study are included within the article.
